# Global Research Trends and Healthcare Innovations in Plantar Pressure Management for Diabetic Foot Ulcers: A 25-Year Bibliometric and Visual Analysis

**DOI:** 10.3390/healthcare14060780

**Published:** 2026-03-19

**Authors:** Dehua Wei, Boya Li, Jiangning Wang, Lei Gao

**Affiliations:** Orthopedic Department, Capital Medical University Affiliated Beijing Shijitan Hospital, No. 10 Yangfangdian Tieyi Road, Haidian District, Beijing 100038, China; weidehua6290@bjsjth.cn (D.W.); liboya@bjsjth.cn (B.L.); wangjn@bjsjth.cn (J.W.)

**Keywords:** diabetic foot ulcers, plantar pressure, bibliometric analysis, CiteSpace, VOSviewer, foot biomechanics, offloading, wound healing, research trends, co-citation analysis

## Abstract

**Background:** Diabetic foot ulcers (DFUs) represent a major chronic complication of diabetes mellitus, often leading to severe infection, amputation, and reduced quality of life. Among various factors affecting DFUs, plantar pressure plays a pivotal role in ulcer formation and recurrence. Despite growing interest in this domain, few studies have comprehensively evaluated the research landscape concerning plantar pressure in the context of DFUs from a bibliometric perspective. **Aim:** To conduct a comprehensive bibliometric analysis and visualization of global research trends, hotspots, and collaborative networks in the field of plantar pressure-related diabetic foot studies from 2000 to 2024. **Methods:** A systematic search was conducted in the Web of Science Core Collection (WoSCC) on 16 February 2025, for articles published between 2000 and 2024 using terms related to “diabetic foot” and “plantar pressure”. A total of 2518 records were retrieved, from which 2110 English-language articles and reviews were included. Bibliometric and visual analyses were performed using Microsoft Excel 2021, VOSviewer (v1.6.20), CiteSpace (v6.4.R1), Charticulator, and Scimago Graphica. Analyses included publication trends, country/institution/author collaborations, journal distributions, keyword co-occurrence and clustering, citation bursts, and reference co-citation networks. **Results:** A total of 2110 publications were identified, showing an overall increase in annual publication output from 2000 to 2024, with some year-to-year fluctuations. The United States led in publication volume (678 articles), citation frequency, and H-index, followed by the United Kingdom and China. Armstrong, David was the most prolific and also had the highest H-index among the listed authors, while the University of Amsterdam was the leading institution. “Journal of Wound Care” had the highest publication count, whereas “Diabetes Care” ranked first in citation frequency. Keyword analysis revealed major research clusters including “diabetic foot”, “plantar pressure”, “wound healing”, “offloading”, and “negative pressure wound therapy”. Recent trends show an increased focus on microcirculation, regenerative medicine, customized footwear, and wound care technologies. **Conclusions:** The bibliometric analysis reveals research trends and current hotspots in plantar pressure management for diabetic foot ulcers, with a particular focus on managing plantar pressure through personalized offloading strategies and custom footwear. These findings highlight the practical value of tailoring interventions to individual patient needs, emphasizing the importance of biomechanical factors in ulcer prevention and healing.

## 1. Introduction

Diabetic foot ulcers (DFUs) are among the most severe and common complications of diabetes mellitus and represent the leading cause of lower limb amputations worldwide [1]. Approximately 19–34% of individuals with diabetes are estimated to develop foot ulcers during their lifetime [2,3]. The development and healing of DFUs are influenced by a combination of factors, including neuropathy, peripheral arterial disease, local biomechanical stress, and infection [4]. Notably, abnormal plantar pressure distribution has been identified as a critical mechanical contributor to both the initiation and recurrence of foot ulcers in patients with diabetes [5].

Plantar pressure reflects the mechanical load on different regions of the foot during gait and standing. Increased forefoot and hallux pressure, along with reduced mobility and altered pressure redistribution, are closely associated with ulcer formation, particularly in patients with peripheral neuropathy who lack protective sensation [6]. Clinical interventions such as offloading footwear, total contact casting, and custom-made insoles aim to alleviate localized pressure and prevent ulceration [7]. Despite significant advances in therapeutic techniques, the recurrence rate of healed DFUs remains high, largely due to unresolved biomechanical risk factors [8].

Bibliometrics is a statistical and quantitative method that enables the systematic analysis of the published academic literature within a particular field [9]. It is widely used to explore the knowledge structure, research hotspots, collaboration networks, and development trajectories of a given research domain. Tools such as CiteSpace (v6.4.R1), VOSviewer (v1.6.20), and Scimago Graphica have been developed for visualizing co-authorship, co-citation, and keyword clustering, thereby facilitating the identification of emerging trends and research frontiers [10]. Bibliometric approaches have been extensively applied in various medical disciplines, including wound healing, orthopedics, and diabetic complications [11]. The development of diabetic foot ulcers is closely linked to abnormal plantar pressure, yet there are virtually few bibliometric studies examining this relationship. Existing reviews primarily focus on pathological mechanisms and clinical treatments, lacking a systematic summary of research hotspots, collaboration networks, and development trends related to plantar pressure. Therefore, this study aims to provide a descriptive bibliometric mapping of publication patterns, hotspots, and collaborative structures in plantar pressure-related DFU research.

## 2. Materials and Methods

### 2.1. Data Source and Search Strategy

To identify relevant publications related to plantar pressure in diabetic foot research, a comprehensive literature search was performed in the Web of Science Core Collection (WoSCC) on 16 February 2025. It was selected as the primary data source because it is considered the most prestigious database for bibliometric studies, providing robust citation data and comprehensive metadata. The following search strategy was used: TS = (“diabetic foot” OR “foot, diabetic” OR “diabetic feet” OR “feet, diabetic” OR “footulcer, diabetic” OR “diabetic foot ulcer” OR “DFUs”) AND TS = (“Foot plantar pressure” OR “Plantar pressure” OR “Pressure”). The inclusion criteria were restricted to original articles and reviews written in English, published between 1 January 2000 and 31 December 2024. The data source was limited to the Science Citation Index Expanded (SCIE) to ensure scientific rigor and standardization. A total of 2518 records were initially retrieved. Given the interdisciplinary nature of DFU care, this study defined the target literature as DFU publications linked to plantar pressure, pressure redistribution, or pressure management contexts, rather than restricting the corpus to biomechanics-only studies. To ensure a strict focus on DFUs, titles and abstracts were reviewed to exclude non-diabetic or non-foot-related pressure studies. Additionally, a thesaurus was applied to merge synonymous terms and filter out irrelevant keywords. After applying language and document type filters, 2110 valid articles and reviews were included in the final analysis. Literature screening and data downloading were completed on the same day to reduce variability caused by database updates.

Two investigators independently performed the literature retrieval and screening procedures. Any discrepancies identified during the screening process were resolved through discussion until consensus was reached.

### 2.2. Data Collection and Cleaning

The original dataset extracted from the WoSCC included multiple bibliometric parameters: publication year, document type, journal title, authorship, institutions, countries/regions, citation frequency, H-index, keywords, and references. Manual preprocessing was performed to correct misspellings, unify author names, and eliminate duplicate records. A thesaurus file was constructed to consolidate synonymous terms, correct institution variants, and unify keywords (e.g., “diabetic foot ulcer” and “DFU”). Although minor errors related to author name duplication, citation variations, and journal abbreviation inconsistencies were unavoidable, the majority of the data were considered reliable after manual curation.

### 2.3. Bibliometric Analysis

**Figure 1:**
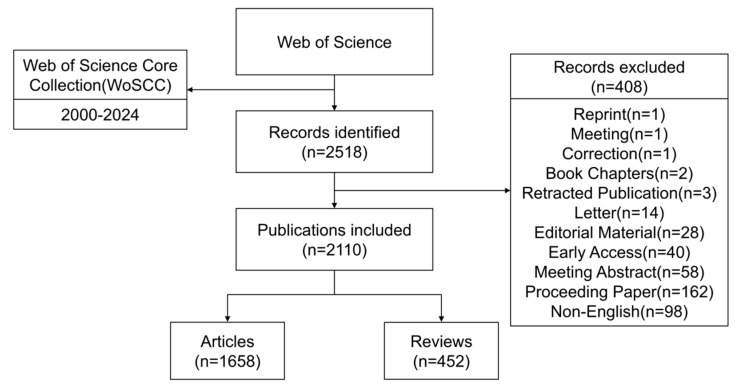
Flowchart of literature search, selection and bibliometric analysis.

Multiple software tools were utilized to perform quantitative and visual bibliometric analyses; HistCite (v12.03.07) was employed to determine the annual publication output, document types, and overall citation trends. Microsoft Excel 2021 was used for data collation and descriptive visualization of publication patterns. The present study is a descriptive and exploratory bibliometric analysis intended to map publication characteristics, collaboration structures, and research hotspots, rather than to perform inferential statistical modeling or validated forecasting. VOSviewer (v1.6.20) was used to analyze co-authorship, institutional and country collaboration networks, keyword co-occurrence, and citation mapping. Full counting was selected as the counting method. Custom threshold values (T) were set according to analysis objectives; for example, a minimum of 5 publications for countries/regions, and 10 citations for journals/authors. CiteSpace (v6.4.R1) was applied to detect emerging keywords, conduct burst detection, and generate clustering of keyword co-occurrence networks. Parameters included time slicing (2000–2024), one-year per slice, and pruning algorithms (pathfinder, pruning sliced networks). Scimago Graphica (v1.0.36.0) was used for advanced network visualization, particularly geographic and institutional publication maps. In VOSviewer-generated maps, nodes represent elements such as countries, institutions, or keywords, with node size reflecting frequency or citation count. The lines between nodes indicate co-occurrence or collaboration links, and the thickness of the lines corresponds to their strength. The CiteSpace visualizations further supported trend detection and highlighted key turning points in the research evolution. A flowchart summarizing the bibliometric methodology is provided in Figure 1.

## 3. Results

### 3.1. Publication Trends and Subject Categories

**Figure 2:**
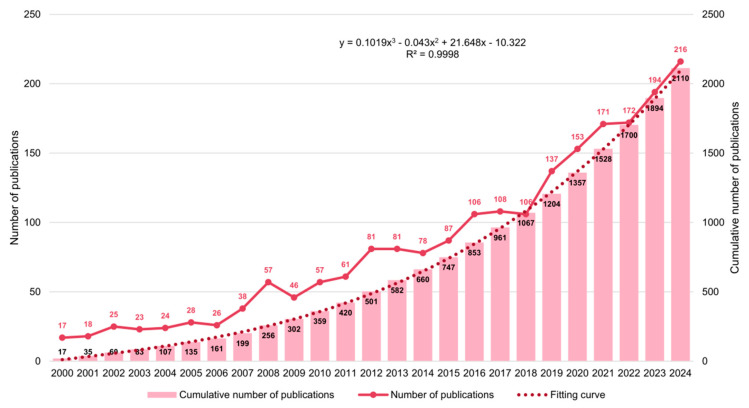
Annual publication output from 2000 to 2024. The red dashed line in Figure 2 is presented only as a visual smoothing/reference line to aid pattern recognition and should not be interpreted as a validated inferential or forecasting model.

The annual number of publications is presented as a descriptive indicator of research activity in this field. As shown in Figure 2, annual scientific output related to plantar pressure in diabetic foot research generally increased from 2000 to 2024, with intermittent fluctuations across years. To avoid overinterpretation, no inferential curve-fitting or forecasting model was used to support trend claims. If a fitted line is displayed in the figure, it is used only as a visual aid to summarize the overall pattern rather than as evidence for statistical prediction. These findings indicate sustained research attention to this topic during the study period (2000–2024).

A total of 2110 articles were categorized across 101 Web of Science research areas, reflecting the interdisciplinary nature of the field. Table 1 presents the top 20 most represented subject categories. The category with the highest number of publications was Surgery, encompassing 522 articles, followed closely by Dermatology (501 articles), Endocrinology and Metabolism (304), Orthopedics (241), and Medicine, General and Internal (176). These findings highlight the central role of surgical and dermatological approaches in diabetic foot research, while also demonstrating significant contributions from endocrinology, musculoskeletal medicine, and general internal medicine, suggesting a multidisciplinary convergence in addressing plantar pressure-related diabetic foot pathology.

### 3.2. Countries/Regions: Volume, Impact, and Collaboration

**Figure 3:**
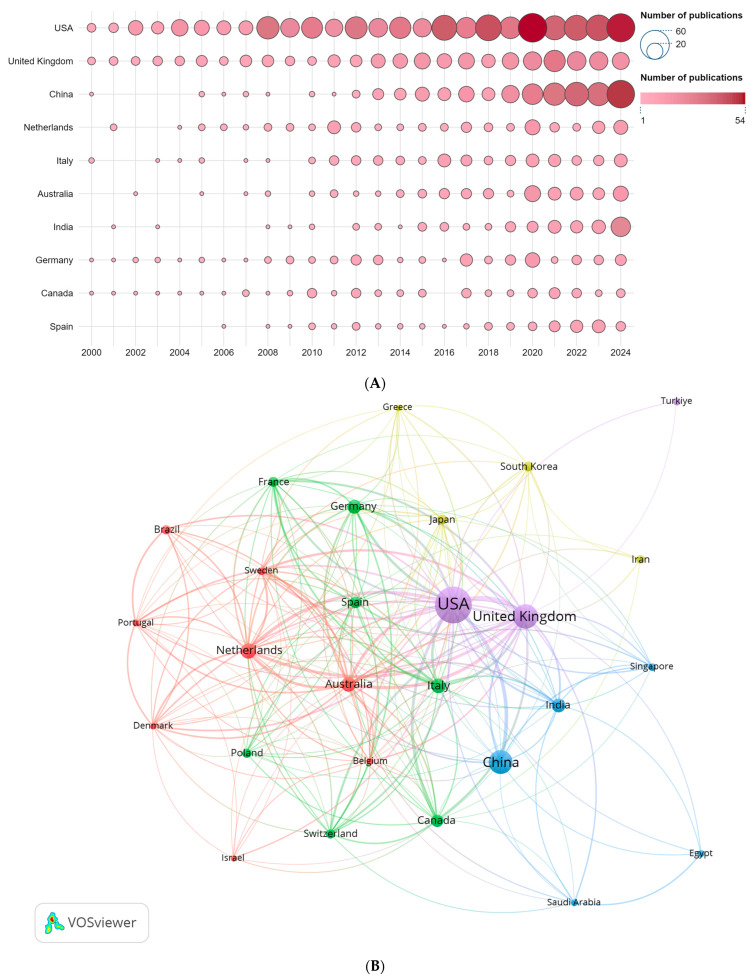

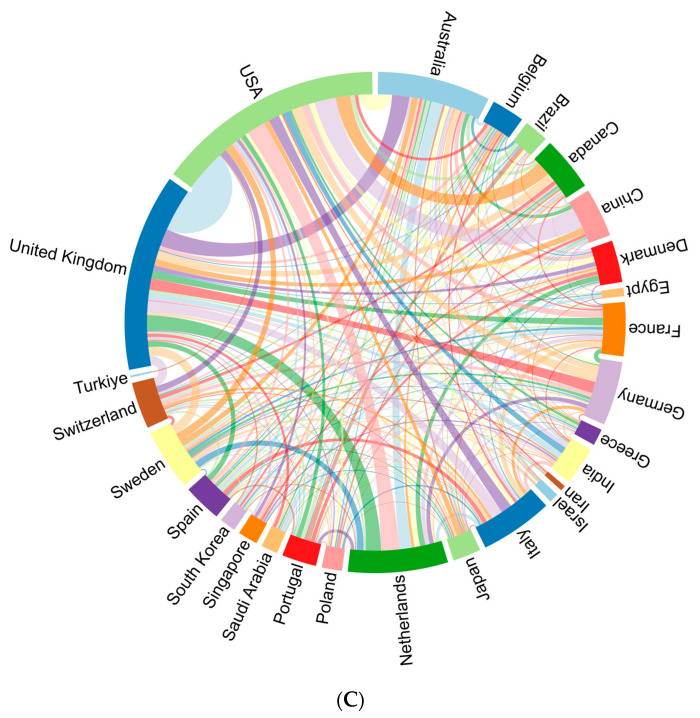
(**A**) Bubble plot of publication volume by country/region. (**B**) International collaboration network among countries/regions. (**C**) Country/region collaboration chord diagram.

Globally, 90 countries/regions have contributed to this field. Table 2 lists the top ten by publication volume. The United States (USA) produced the largest output (n = 678). The Netherlands achieved the highest average citations per article, while the United Kingdom, the USA, and Canada also showed relatively high citation impact per publication; by contrast, India and Spain showed lower averages, suggesting scope to enhance visibility and quality. The USA also recorded the highest H-index, underscoring its leading influence in this domain. Figure 3A depicts annual outputs for the top ten countries/regions: the United States has maintained long-standing leadership, rising from five publications in 2000 to 52 in 2024, with pronounced peaks in 2008 (n = 33), 2016 (n = 41), and 2020 (n = 54), reflecting sustained research competitiveness. China increased from one publication in 2000 to 50 in 2024, with accelerated growth after 2015 (e.g., 33 in 2021, 50 in 2024), indicating strong investment and translation of research outputs. The United Kingdom showed an overall upward yet fluctuating trajectory, peaking at 30 publications in 2021 before declining to 19 in 2024. Among emerging contributors, India reached 25 publications in 2024, representing more than a tenfold increase from two publications in 2010, illustrating notable catch-up momentum. Overall, the global research landscape remains dominated by Europe and North America, while the rise of China, India, and others is gradually reshaping the competitive balance.

Using VOSviewer to analyze inter-country collaborations (displaying only countries/regions with ≥20 publications; n = 27), Figure 3B visualizes the network in which node size reflects publication volume and edges denote collaborative links. To more clearly depict the overall and pairwise collaboration intensity, we further constructed a country-level chord diagram (Figure 3C), where node size encodes a country’s total number of collaborations and edge thickness represents the frequency of bilateral cooperation. The USA and United Kingdom exhibit the most extensive collaborative footprints, indicating high recognition and centrality within the field; notably, the USA–United Kingdom dyad shows the most frequent bilateral collaboration, highlighting their particularly close partnership.

### 3.3. Authors: Productivity, Influence, and Networks

A total of 9307 authors have contributed to this field. Table 3 lists the top ten by publication volume. The most prolific author is David G. Armstrong (76 publications), followed by Sicco A. Bus (52) and Lawrence A. Lavery (40). Benjamin A. Lipsky has the highest average citations per article, indicating strong per-paper quality and recognition within the field. Other highly cited authors include David G. Armstrong, Andrew J. M. Boulton, and Sicco A. Bus. Armstrong also holds the highest H-index, underscoring his substantial influence in this domain.

**Figure 4:**
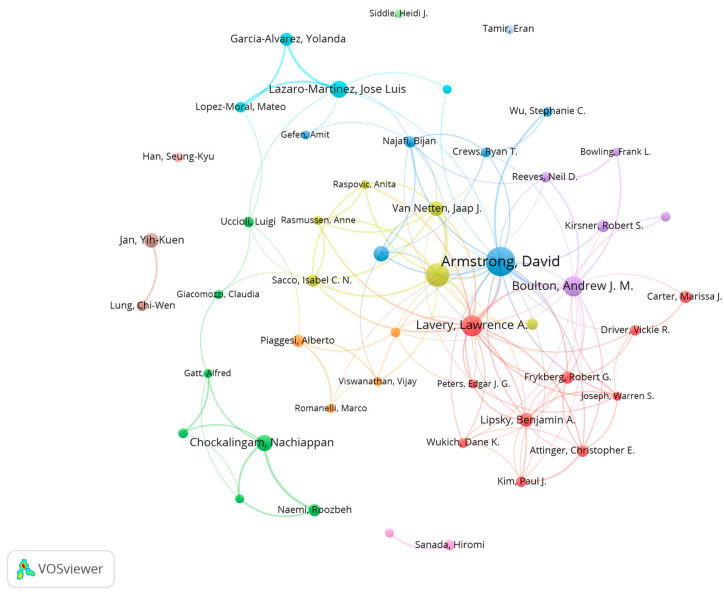
Author co-authorship network.

Using VOSviewer to analyze authors with ≥8 publications (n = 49), the Figure 4 co-authorship network reveals collaboration patterns across research clusters. Key nodes include leading scholars such as David G. Armstrong, Andrew J. M. Boulton (associated with the International Working Group on the Diabetic Foot), and Jaap J. van Netten (expert in foot biomechanics and plantar pressure research), whose close collaborations form the network’s core clusters. In addition, Stephanie C. Wu and Robert S. Kirsner bridge subdomains—linking plantar pressure analysis with wound repair research—through cross-team collaborations. Notably, the comparatively independent nodes of Alberto Piaggesi (Italy) and Hiromi Sanada (Japan) may reflect distinctive regional contributions. Overall, the network exhibits a multi-hub architecture in which core authors drive interdisciplinary integration through high-frequency collaboration; for example, links between Lawrence A. Lavery and biomechanics expert Nachiappan Chockalingam illustrate the convergence of clinical medicine and engineering.

### 3.4. Institutions: Contributions and Collaboration Structure

**Figure 5:**
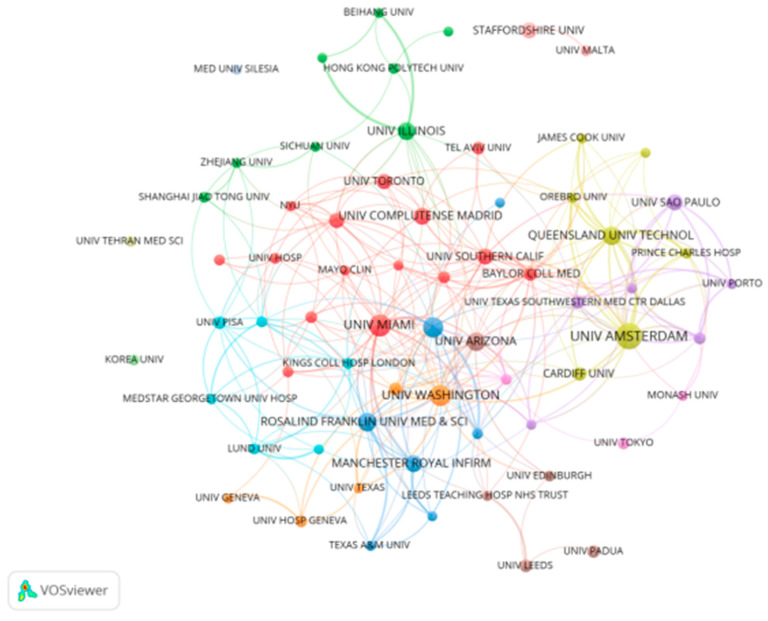
Inter-institutional collaboration network.

A total of 3316 institutions have conducted research in this field. Table 4 lists the top ten institutions by publication volume. The University of Amsterdam ranks first with 68 publications, followed by the University of Miami (46), the University of Washington (43), and the University of Manchester (43). In terms of average citations per article, the University of Washington ranks first among the listed institutions. The University of Amsterdam holds the highest H-index among the listed institutions, indicating a high level of citation impact within this dataset. Figure 5 displays the inter-institutional collaboration network for institutions with ≥10 publications (n = 67), revealing close partnerships that are conducive to the field’s advancement.

### 3.5. Journals and Knowledge Flows

Table 5 (left panel) lists the top ten source journals by publication volume. The United Kingdom’s Journal of Wound Care (IF = 1.5) published the most articles (n = 115), followed by the United Kingdom’s International Wound Journal (IF = 2.6) and the USA’s Wounds—A Compendium of Clinical Research and Practice (IF = 1.4), with 79 and 73 publications, respectively. These patterns indicate that these outlets are especially favored by researchers in this field and have made prominent contributions to its development. Among the ten journals, the highest impact factor belongs to the USA’s Diabetes Care (IF = 14.8). Table 5 (right panel) presents the top ten cited journals by citation frequency. The USA’s Diabetes Care (IF = 14.8) ranks first with 7044 citations, followed by the USA’s Wound Repair and Regeneration (IF = 3.8) and the United Kingdom’s Diabetes/Metabolism Research and Reviews (IF = 4.6), with 2434 and 2374 citations, respectively. This distribution underscores the substantial influence of these journals within the field. Notably, Diabetes Care again has the highest impact factor (IF = 14.8) among these ten journals. Published by the American Diabetes Association (ADA) since 1978, Diabetes Care is an authoritative journal dedicated to clinical research, therapeutic advances, and practice guidelines in diabetes. Its scope spans prevention, diagnosis, treatment, and complication management; it is particularly recognized for annually publishing the ADA Standards of Medical Care in Diabetes, which guide clinical practice worldwide. Article types include original investigations, reviews, guidelines, and cross-disciplinary studies, with emphases on drug innovation, glucose-monitoring technologies, and the broader landscape of metabolic diseases.

**Figure 6:**
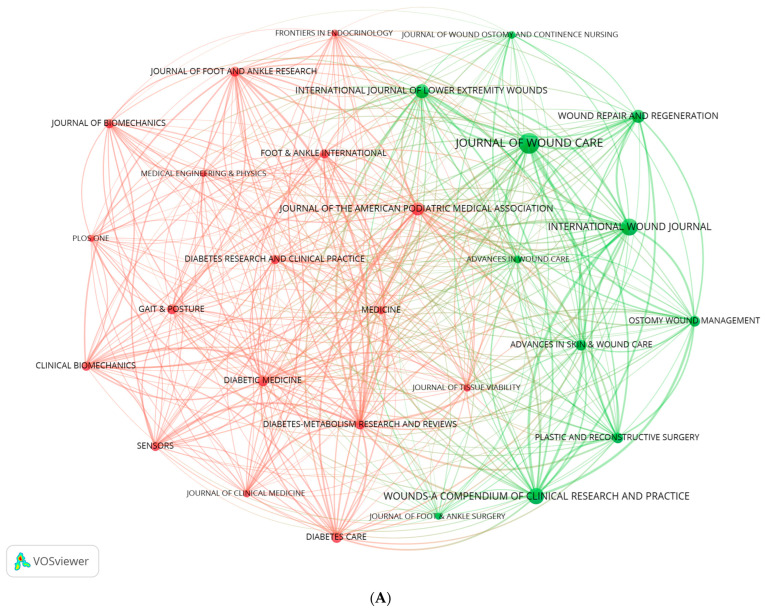

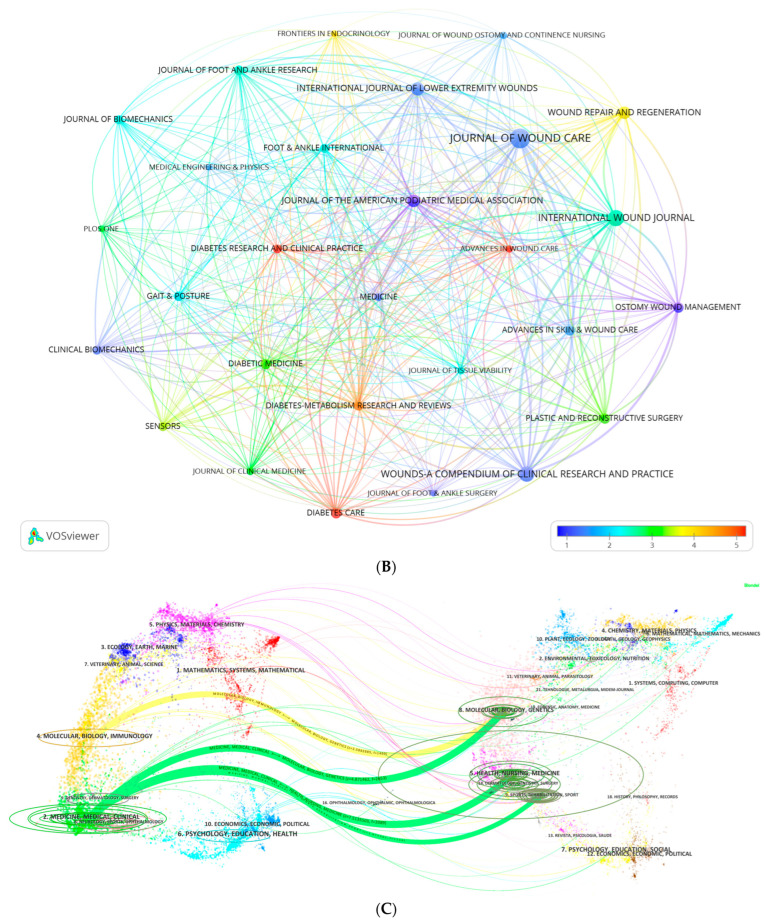
(**A**) Journal co-citation network (coupling analysis). (**B**) Impact factors overlaid on the journal co-citation network. (**C**) Dual-map overlay of source and cited journals.

Figure 6A depicts the journal co-citation network (coupling analysis). Nodes represent journals with ≥15 publications (n = 28); node size reflects publication volume, and edges indicate instances in which two journals co-cite the same article. Figure 6B overlays impact factors onto the co-citation network and encodes them by color, with blue indicating lower impact factors and red indicating higher ones. Figure 6C presents the dual-map overlay of source (left) and cited (right) journals for the 2110 included articles. Source journals reflect application domains, whereas cited journals represent foundational disciplines. Colored trajectories denote citation paths across domains, visualizing inter-field relationships and knowledge flow at the journal level. Using the software’s built-in Z-score algorithm, four knowledge flow paths were identified; colors correspond to regions on the cited side, and line width is proportional to the cited Z-score. The principal citation streams originate from “MOLECULAR, BIOLOGY, GENETICS”, “HEALTH, NURSING, MEDICINE”, and “SPORTS, REHABILITATION, SPORT”, converging on frontier areas such as “MOLECULAR, BIOLOGY, IMMUNOLOGY” and “MEDICINE, MEDICAL, CLINICAL”. Of note, the path from “HEALTH, NURSING, MEDICINE” to “MEDICINE, MEDICAL, CLINICAL” exhibits the most prominent Z-value (z = 7.514), underscoring its importance and impact.

### 3.6. Keyword Landscape and Research Hotspots

Using the g-index selection criterion with k = 10, we initially extracted 271 keywords from the 2110 included articles. Synonymous terms were merged and non-informative terms that did not convey meaningful content for this study were removed, yielding 29 merged keywords and six deletions. After creating the merge and delete thesaurus files, we re-ran the analysis with k = 5 (to avoid the introduction of new terms) and identified 153 keywords. The finalized results were then visualized.

**Figure 7:**
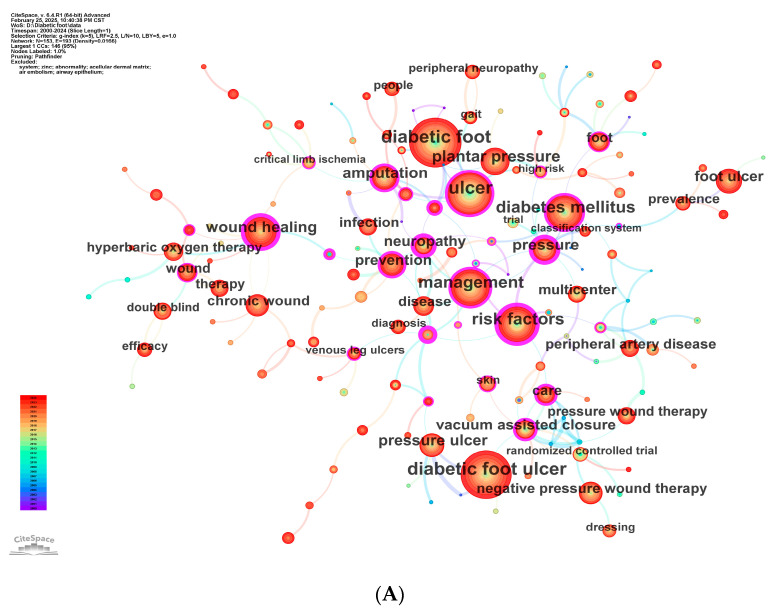

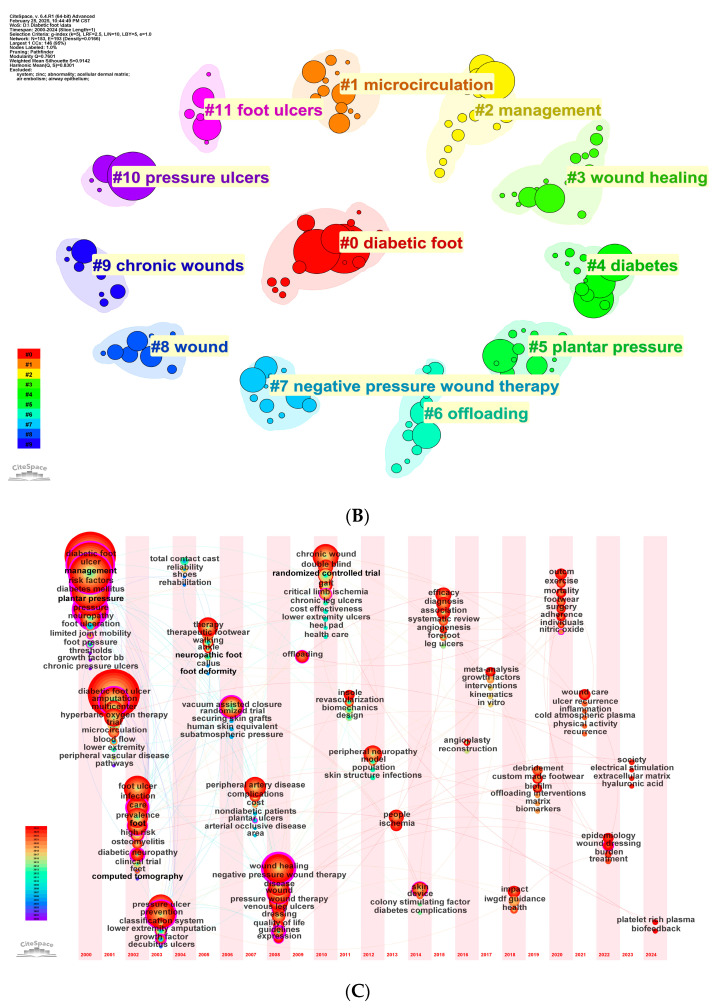
(**A**) Keyword co-occurrence network. (**B**) Keyword clustering. (**C**) Keyword time zone map.

Figure 7A presents the keyword co-occurrence network. Nodes represent keywords; node size encodes occurrence frequency (larger nodes = higher frequency). Node color transitions from blue (earlier years) to red (recent years), reflecting the recency of appearances; the color legend by year is shown in the lower-left inset. Each node contains annual ring bands whose band width reflects the keyword’s frequency in that specific year. A purple outer ring indicates betweenness centrality > 0.1, i.e., a pivotal bridging role in the network. Edges connect keyword pairs co-appearing in the same article; thicker edges denote more frequent co-occurrence. Labels at node centers are the keywords themselves, and label font size scales with frequency.

High-frequency terms—“diabetic foot” (freq 633) and “diabetic foot ulcer” (freq 609)—occupy central positions, underscoring their foundational status. Despite modest frequencies, “risk factors” (freq 378, centrality 0.54) and “guidelines” (freq 28, centrality 0.55) show high centrality, functioning as network hubs that interlink management, infection, and neuropathy subdomains; notably, the strong tie between “management” and “risk factors” highlights their central role in prevention and care pathways. The tight co-occurrence of “ulcer” (freq 520, centrality 0.22) with “diabetes mellitus” (freq 364, centrality 0.25) corroborates the close coupling between ulceration and metabolic dysregulation. By contrast, “plantar pressure” (freq 290, centrality 0.06) is frequent but exhibits low centrality, suggesting a focus on local biomechanics with limited cross-domain bridging.

Building on the co-occurrence map in Figure 7A, Figure 7B presents keyword clustering performed in CiteSpace using the log-likelihood ratio (LLR) algorithm, with cluster labels extracted from keyword fields. In total, 12 clusters were generated. The modularity (Q) = 0.7601 (>0.3) indicates a salient cluster structure, and the silhouette (S) = 0.9142 (>0.7) supports highly convincing clustering. The clusters are: #0 diabetic foot, #1 microcirculation, #2 management, #3 wound healing, #4 diabetes, #5 plantar pressure, #6 offloading, #7 negative pressure wound therapy, #8 wound, #9 chronic wounds, #10 pressure ulcers, and #11 foot ulcer. Based on cluster membership, the thematic implications are summarized below.

Cluster 0 (Core complications and treatment in diabetic foot)

Anchored by high-frequency terms “diabetic foot” (633; centrality 0.09) and “ulcer” (520; centrality 0.22), and encompassing “amputation” (222; centrality 0.29) and “diabetic neuropathy” (34; centrality 0.30), alongside localized interventions such as “surgery” (17) and “callus” (2). This cluster captures the clinical deterioration trajectory from ulceration to amputation. The conjunction of high frequency and high centrality underscores its dual role as both a research hotspot and a cross-domain hub linking mechanisms and interventions.

Cluster 1 (Vascular pathology and microcirculation management)

Focused on “peripheral artery disease” (121; centrality 0.04) and “microcirculation” (27; centrality 0.01), integrating device-based measures such as “therapeutic footwear” (31) and “total contact cast” (17), and terminal outcomes like “lower extremity amputation” (29). Despite moderate frequencies, the pathophysiological chain—from microcirculatory impairment to amputation—is coherent, highlighting the prognostic weight of the vascular microenvironment and the need for deeper cross-disciplinary integration.

Cluster 2 (Comprehensive management and health outcomes)

Organized around high-centrality nodes “management” (392; centrality 0.25) and “guidelines” (28; centrality 0.55), bridging “neuropathy” (160; centrality 0.51) and “quality of life” (28). The centrality of “guidelines” positions it as a network hub for standardization and individualized care, reflecting an evolution from routine practice to evidence-based management.

Cluster 3 (Ischemia and regenerative repair technologies)

Centered on “wound healing” (215; centrality 0.40) and “critical limb ischemia” (42; centrality 0.19), linked to “revascularization” (19) and “growth factor” (14; centrality 0.28). Lower-frequency terms such as “extracellular matrix” (5) point to emerging opportunities and challenges at the interface of molecular mechanisms and translational repair strategies.

Cluster 4 (Risk factors and plantar pressure control)

Driven by “risk factors” (378; centrality 0.54) and “diabetes mellitus” (364; centrality 0.25), integrating biomechanical monitoring such as “plantar pressure” (290; centrality 0.06) and “foot pressure” (8). The relatively low centrality of “plantar pressure” suggests a focus on local technical aspects with insufficient linkage to broader clinical outcomes—an area for future integration.

Cluster 5 (Foot biomechanics and modeling)

Structured around “plantar pressure” (290) and “biomechanics” (14), with “walking” (28) and “model” (13) indicating kinetic/kinematic modeling. The dispersion of terms (e.g., “kinematics”, 5) implies limited depth to date and the need for stronger interdisciplinary collaborations.

Cluster 6 (Prevention and offloading interventions)

Combining “prevention” (184; centrality 0.26), “offloading” (36; centrality 0.14), and “custom made footwear” (20). The presence of “IWGDF guidance” (23) underscores the normative role of international guidelines. This cluster emphasizes the continuum from risk stratification to personalized offloading.

Cluster 7 (Therapeutic technology network for wound care)

Centered on “vacuum assisted closure” (156; centrality 0.22) and “negative pressure wound therapy” (134), integrating “dressing” (45) and “randomized controlled trial” (47). Lower-frequency device terms (e.g., “device”, 10) suggest that newer modalities (e.g., platelet-rich plasma) require more rigorous clinical validation.

Cluster 8 (Wound nursing and therapy evaluation)

Organized around “wound care” (28) and “efficacy” (55), linked with “therapy” (102) and novel approaches such as “cold atmospheric plasma” (5). The frequency gradients (e.g., “efficacy” vs. “interventions”) point to an evaluation framework that remains to be fully standardized.

Cluster 9 (Chronic wounds and inflammation control)

Focused on “chronic wound” (142; centrality 0.10), connected to “biofilm” (19; centrality 0.09) and “inflammation” (5). Lower-frequency items such as “hyaluronic acid” (5) indicate early-stage exploration of matrix repair and anti-inflammatory strategies.

Cluster 10 (Ulcer classification and regenerative medicine)

Contrasts “diabetic foot ulcer” (609) with “pressure ulcer” (194), incorporating regenerative techniques such as “reconstruction” (5) and “human skin equivalent” (3). Lower frequencies (e.g., “colony stimulating factor”, 3) suggest that clinical translation and validation remain key bottlenecks.

Cluster 11 (Epidemiology and individualized intervention)

Integrates “foot ulcer” (185), “epidemiology” (20), and “individuals” (10), focusing on population characteristics and personalized approaches (e.g., “plantar ulcers”, 3). The high share of low-frequency terms (e.g., “diabetes complications”, 3) highlights the need to strengthen links between population research and precision care.

Taken together, these clusters partition the field along a four-dimensional framework—complications, pathophysiology, management strategies, and technological innovation. High-frequency terms define the clinical core (e.g., ulcer, amputation), high-centrality terms (e.g., risk factors, guidelines) act as cross-disciplinary bridges, and lower-frequency clusters (e.g., #10, #11) reveal promising directions in regenerative medicine and precision interventions. Future work should further integrate biomechanical modeling, chronic wound immunobiology, and evidence-based evaluation to enhance systematization and translational impact.

In the keyword time zone map (Figure 7C), the x-axis represents the first appearance of each keyword. From 2000 to 2024, the field exhibits distinct stages:

Foundational phase (2000–2005):

Core themes include “diabetic foot ulcer management risk factors” and “diabetes mellitus”, establishing the basis for pathophysiological inquiry. Early emergence of “neuropathy” and “plantar pressure” foreshadows exploratory work on neurovascular–biomechanical interactions.

Clinical technology expansion (2006–2015):

Keywords extend toward device-enabled and evidence-based care—e.g., “total contact cast” and “negative pressure wound therapy”. The rise of “randomized controlled trial” signals a methodological shift toward evidence-based medicine, while “guidelines” catalyze standardized management.

Innovation and refinement (2016–2024):

Cross-disciplinary integration deepens; regenerative medicine advances via “human skin equivalent” (2018) and “growth factor” (2020); precision focuses on “biofilm” (2019) and “microcirculation” (2021); evidentiary strength is reinforced by “vacuum assisted closure” (2016) in conjunction with “randomized trial” (2022); and emerging modalities such as “cold atmospheric plasma” (2023) enter exploration. Personalized prevention is refined by “custom made footwear” (2020) and “offloading interventions” (2021). Recent entries—“extracellular matrix” (2022) and “hyaluronic acid” (2023)—point to convergence between matrix biology and precision therapeutics.

Burst detection helps track shifting hotspots, current foci, and future directions. Table 6 lists the top 25 burst terms. In the early period (2000–2005), high-strength bursts such as “neuropathy” (16.1; 2000–2008) and “ulcer” (14.02; 2000–2006) framed foundational mechanisms around neuropathy, ulceration, and risk. The mid-period (2006–2015) pivots to clinical techniques and evidence generation—“total contact cast” (8.26; 2004–2013), “trial” (9.1; 2007–2015), and “multicenter” (5.68; 2009–2017)—reflecting standardized, multi-site RCTs. Recently (2016–2024), bursts have become more refined and system-oriented; “iwgdf guidance” (11.33; 2018–2020) underscores the normative role of international guidelines; “microcirculation” (7.63; 2019–2021) and “growth factors” (5.75; 2017–2019) deepen mechanistic and regenerative lines; “custom made footwear” (6.07; 2019–2020) and “wound care” (9.09; 2021–2024) consolidate personalized prevention and care pathways. The emergence of “epidemiology” (8.17; 2022–2024) and “wound dressing” (6.95; 2022–2024) signals a tilt toward population-level management and technological innovation. High-intensity, short-cycle topics (e.g., “debridement”) should be translated into sustained research programs to address the systemic complexity of DFU pathobiology.

### 3.7. Co-Citation Structure and Reference Bursts

Using the g-index (k = 25), we extracted top-ranked references from the 2110 included articles and identified 1286 unique cited references. These references were subjected to co-citation and clustering analyses to delineate the field’s core knowledge base and the evolution of research hotspots.

**Figure 8:**
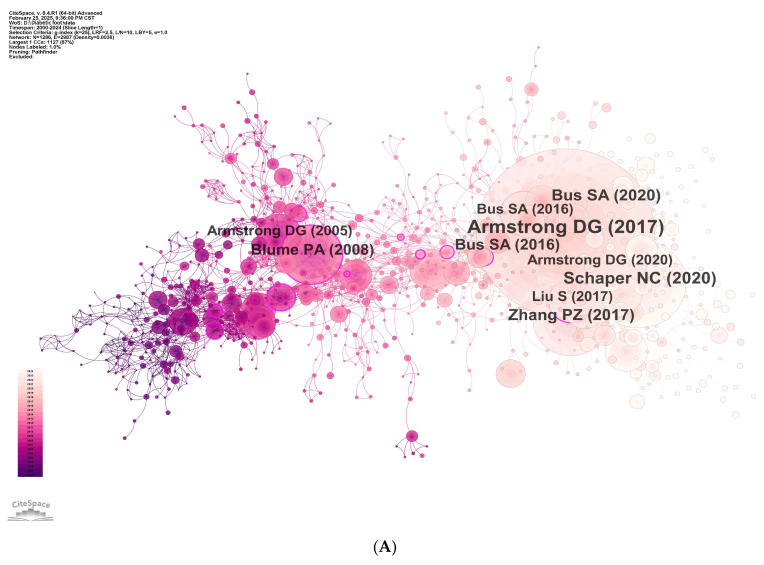

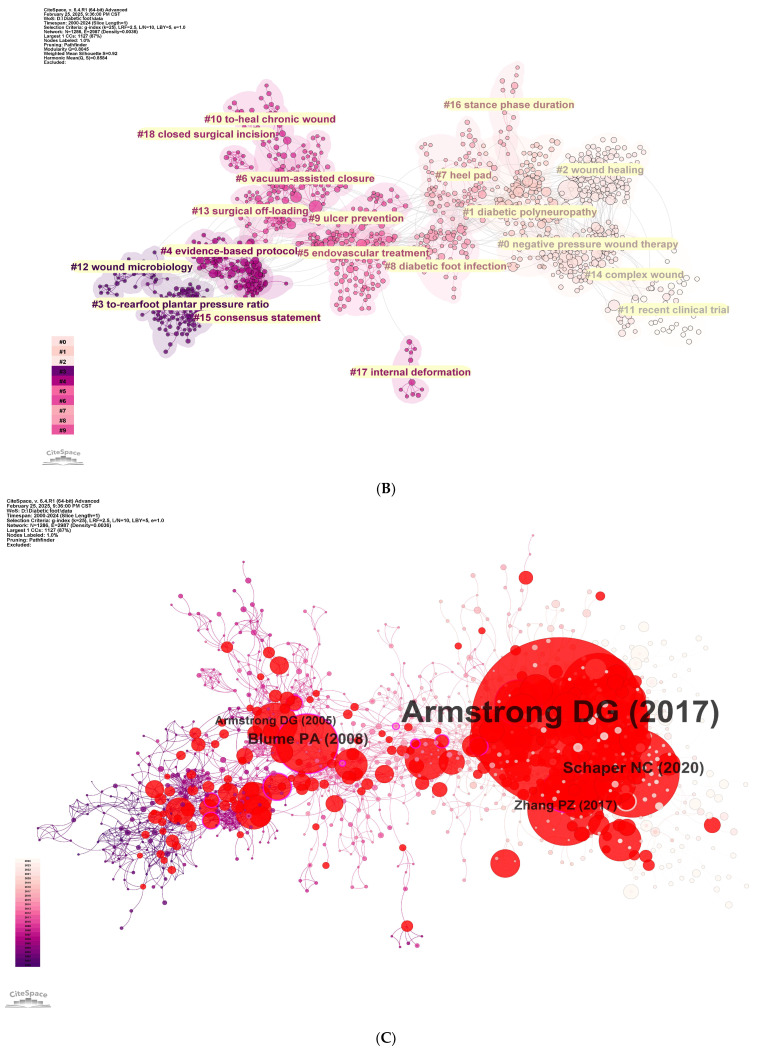
(**A**) Reference co-citation network. (**B**) Reference co-citation clusters. (**C**) References with citation bursts [1,2,12,13,14].

Figure 8A displays the reference co-citation network, with labels for the ten most highly cited items (first author and year). The node representing Armstrong, David G., 2017, The New England Journal of Medicine, “Diabetic Foot Ulcers and Their Recurrence” is the largest, with the highest co-citation count (n = 150), indicating the strongest influence and broad uptake by subsequent studies. That article synthesizes epidemiology, pathophysiology, clinical management, and prevention of diabetic foot ulcers (DFUs); it reports an annual incidence of 2.2–6.3% and a 5-year mortality approximately 2.5-fold that of people with diabetes without ulcers; >50% of ulcers may become infected and about 20% of moderate-to-severe infections may culminate in amputation. Although most ulcers can heal with debridement, offloading, antimicrobial therapy, and revascularization (≈77% within one year), recurrence remains substantial (40% at one year; 65% at five years). The authors therefore advocate framing “healed” as “remission” and emphasize recurrence prevention via continuous professional foot care, custom offloading footwear, patient education to improve adherence, biomechanical corrective surgery, and skin temperature monitoring for early warning. By integrating global epidemiology, clinical studies, and meta-analyses, cross-referencing multiple IWGDF guidelines, and popularizing the remission concept, the paper has reshaped clinical practice and consequently attracts extensive citations.

Building on this, LLR-based clustering (labels extracted from titles) yielded 19 major clusters with Q = 0.8045 (>0.3) and S = 0.92 (>0.7), indicating a salient and convincing structure (Figure 8B): #0 negative pressure wound therapy; #1 diabetic polyneuropathy; #2 wound healing; #3 to-rearfoot plantar pressure ratio [sic]; #4 evidence-based protocol; #5 endovascular treatment; #6 vacuum-assisted closure; #7 heel pad [sic]; #8 diabetic foot infection; #9 ulcer prevention; #10 to-heal chronic wound [sic]; #11 recent clinical trial; #12 wound microbiology; #13 surgical off-loading; #14 complex wound; #15 consensus statement; #16 stance phase duration; #17 heel pad [sic]; and #18 closed surgical incision.

Reference bursts reflect items with rapidly increasing attention over short intervals; greater burst strength indicates stronger period-specific influence and helps surface emerging frontiers. Figure 8C overlays burst references on the co-citation map from Figure 8A (red nodes), with labels marking the top five by burst strength. The strongest burst again corresponds to Armstrong et al., 2017 [2], The New England Journal of Medicine. Table 7 lists the top 25 burst references; notable high-strength bursts that persist to the present include “Practical Guidelines on the Prevention and Management of Diabetic Foot Disease (IWGDF 2019 update)”, “Guidelines on the Prevention of Foot Ulcers in Persons with Diabetes (IWGDF 2019 update)”, and “Five-year mortality and direct costs of care for people with diabetic foot complications are comparable to cancer”.

## 4. Discussion

### 4.1. Annual Growth Trend Analysis

Over the past quarter-century, research on plantar pressure in diabetic foot has expanded exponentially, mirroring the rising worldwide burden of diabetic foot ulcers (DFUs). In our bibliometric analysis, annual publications grew almost linearly from 2000 to 2024 (R^2^ ≈ 0.9998), indicating sustained and accelerating interest. This trend aligns with epidemiologic data showing DFUs affect approximately 18.6 million people globally each year [34,35].

The dire prognosis associated with DFUs has galvanized multidisciplinary interest; notably, 5-year mortality in patients with a healed DFU approaches 30–50%, rivaling many malignancies [35]. In response, the annual volume of DFU plantar pressure publications increased nearly linearly from 2000 to 2024 in our analysis, reflecting sustained scientific and clinical engagement. High-risk populations in North America and Europe have driven much of the literature historically, with the United States and United Kingdom producing the most citations and foundational work [2]. Classic studies from these regions established fundamental concepts—for example, the landmark recognition that a DFU is often the “pathway to amputation” and not a benign event [3]. In recent years, emerging economies with large diabetes populations (e.g., China and India) have markedly expanded their contributions, diversifying the evidence base [36]. This globalization of scholarship is further evidenced by internationally co-authored consensus reports and guidelines. Experts from multiple continents routinely collaborate on guidance documents such as the International Working Group on the Diabetic Foot (IWGDF) guidelines, helping to unify terminology and standards of care [14]. These collaborations have produced comprehensive, evidence-based recommendations covering prevention, offloading, wound management, and infection control in DFUs [37]. The net effect is a more interconnected research community and a shared global strategy to address DFUs. Nonetheless, disparities remain; while some high-income countries report declining major amputation rates due to concerted foot care programs, many regions still lack access to multidisciplinary teams and offloading resources [38]. Bridging these gaps through international partnerships and knowledge transfer is an ongoing priority. Overall, the literature trends underscore both a quantitative growth in research output and a qualitative shift toward collaboration and consensus-building across disciplines and borders, laying a strong foundation for the next generation of diabetic foot innovations [39]. Bibliometric trends highlight the growing importance of plantar pressure in diabetic foot ulcer (DFU) research. Key clinical innovations focus on personalized offloading strategies, such as customized footwear and 3D printed insoles, to manage abnormal plantar pressure. These approaches aim to reduce localized pressure, prevent ulcer formation, and improve healing outcomes. However, despite significant advances, challenges remain in fully integrating biomechanical insights into broader clinical care. Future research should focus on refining these interventions, exploring the intersection of biomechanics and DFU recurrence prevention, and optimizing personalized care strategies.

### 4.2. Biomechanical Risk Factors and Plantar Pressure Mechanisms

An enduring theme in the DFU literature is the causal role of abnormal biomechanics—especially elevated plantar pressures—in ulcer development. Early studies by Boulton and colleagues first demonstrated in the 1980s that patients with diabetic neuropathy exhibit abnormal plantar pressure distributions and loss of protective sensation, creating a setup for repetitive pressure injuries [40]. Subsequent studies established the classic triad of peripheral neuropathy, foot deformity, and focal high pressure as the primary pathway to plantar ulceration [41]. In neuropathic feet, intrinsic muscle atrophy and joint limitations lead to deformities (e.g., claw toes, prominent metatarsal heads) that concentrate weight-bearing on atypical areas of the sole [42]. During gait, these areas experience elevated peak plantar pressures far above normal levels, particularly under the forefoot and hallux [43]. Prospective risk factor studies have confirmed that higher baseline plantar pressures strongly predict future DFU occurrence [44]. Every 1 kg/cm^2^ increase in forefoot pressure has been associated with a measurable rise in ulcer risk, highlighting pressure as a modifiable risk factor in ulcer prevention [45]. Importantly, it is not only vertical pressure but also shear stress (horizontal frictional force) that contributes to tissue breakdown. A recent systematic review found that patients with a history of DFU exhibit significantly greater plantar shear stresses than diabetics who never ulcerated, even when neuropathy severity is comparable [46]. Yavuz et al. (2014) demonstrated using specialized instruments that shear forces are markedly elevated at common ulcer sites in neuropathic patients, implicating shear as an independent pathogenic factor [47]. These insights have expanded the mechanical paradigm of DFUs beyond simple peak pressure magnitude to a more nuanced understanding of stress distribution and repetitive loading cycles over time [47]. Biomechanical research has also yielded quantitative thresholds for concern. For instance, one cross-sectional study proposed a “critical” barefoot pressure of ~4 kg/cm^2^ at the midfoot above which the likelihood of prior ulceration rose dramatically [48]. Such findings reinforce the concept of identifiable pressure tipping points that could guide clinical screening. In tandem, integrating callus formation into risk assessment is important—callused areas amplify local pressures and often precede ulcers [43]. Longitudinal studies have indeed noted that neuropathic patients with high pressures and callus are at especially high ulceration risk [49]. Taken together, the biomechanical evidence base strongly supports the paradigm that DFUs are largely preventable, mechanically induced injuries superimposed on neuropathic insensitivity. This has spurred development of pressure-based predictive models and risk scoring systems to identify “at-risk” feet before breakdown occurs [50]. It has also informed patient education—for example, teaching patients that areas of redness or callus on the sole signal dangerous pressure points that should be offloaded. In summary, two decades of investigation have cemented plantar pressure as a central etiologic factor in DFU pathogenesis. This body of work provides a scientific rationale for offloading interventions and is directly reflected in the current clinical guidelines, which emphasize routine pressure evaluation and reduction as cornerstone strategies in DFU prevention [51].

### 4.3. Offloading Interventions and Technological Innovation

Recognizing that elevated plantar pressure underpins DFU development and recurrence, a major focus has been devising effective offloading interventions. Therapeutic offloading—redistributing or relieving pressure on weight-bearing areas—is the cornerstone of both DFU prevention and ulcer treatment [52]. Over the past 25 years, numerous modalities have been studied, ranging from specialized footwear and insoles to casts, braces, and surgical corrections. The consensus from clinical trials is that aggressive offloading significantly improves outcomes. For active ulcer treatment, non-removable devices have proven most effective because they ensure adherence to pressure relief [52]. Total contact casting (TCC) is considered the gold standard for healing neuropathic plantar ulcers, yielding healing in roughly 6–8 weeks for uncomplicated ulcers [15]. In a randomized trial, Armstrong et al. (2001) showed TCC healed a higher proportion of ulcers than removable cast walkers or depth shoes, primarily by enforcing constant offloading [53]. Similarly, meta-analyses have confirmed that TCC or non-removable walkers heal ulcers significantly faster than standard care or removable devices [53]. For prevention of ulcers and ulcer recurrence, therapeutic footwear and custom insoles play a critical role. A multicenter trial by Bus et al. (2013) demonstrated that custom-made diabetic footwear (optimized to reduce forefoot pressure by ~20%) reduced 18-month ulcer recurrence by about 50% compared to usual therapeutic shoes [21]. This aligns with earlier findings that adequate offloading can halve the risk of re-ulceration [54]. In Mueller et al.’s study, adding an Achilles tendon lengthening (to increase ankle dorsiflexion and offload the forefoot) resulted in all ulcers healing and a 75% reduction in forefoot ulcer recurrence at 7 months versus casting alone [54]. Such data highlight that surgical offloading procedures (e.g., tendo-Achilles lengthening, metatarsal head resection, corrective osteotomies) can be highly effective adjuncts for patients with recurrent ulcers due to rigid deformities or equinus. Guidelines now recommend considering these surgical interventions in appropriate high-risk cases to address underlying biomechanical deformities [51]. In recent years, technological innovation has driven offloading into the “smart” era. Wearable pressure-sensing systems have been developed to monitor patients’ real-time plantar pressures and alert them to offload when dangerous levels or durations of pressure are detected [55]. One proof-of-concept study provided high-risk patients with an insole that signaled (via smartwatch) when sustained pressure exceeded safe thresholds; adherence to offloading cues improved significantly among those receiving more frequent alerts [55]. Another randomized trial tested an intelligent insole that gave auditory feedback when pressure was too high, and reported a reduction in recurrent plantar ulcers over 18 months in the intervention group [56]. Remote temperature monitoring is another validated approach; by having patients measure foot skin temperature at set sites daily, incipient inflammation (a surrogate for repetitive pressure damage) can be detected and addressed before an ulcer forms [5]. In a landmark multicenter trial, daily temperature self-monitoring plus preventive offloading led to a significant drop in ulcer recurrence (8.5% vs ~30% in standard care)—a nearly 3-fold reduction in risk [5]. This study by Lavery and colleagues established at-home thermometry as an effective adjunct, and subsequent work has confirmed its utility in guiding patients to rest or seek care at the earliest sign of hotspot development [57]. Modern iterations of this concept include smart socks with embedded temperature and pressure sensors, which can transmit data to providers and patients in real time. These technologies underscore a paradigm shift from reactive ulcer care to proactive ulcer prevention using personalized monitoring. However, the uptake of smart offloading devices in practice has encountered some barriers. A recent systematic review of patient and provider perspectives found that while smart insoles and socks are viewed as useful, issues like device comfort, footwear fit, and alert fatigue can limit long-term adherence [58]. Patients in some studies cited the intrusiveness of alerts and the bulk of devices as challenges, even as they acknowledged the value of feedback for behavior change [58]. These insights indicate that further refinement of wearable offloading tech is needed to enhance user acceptability. In parallel, simpler interventions such as patient education and routine podiatry remain important for offloading in daily life—for example, regular debridement of calluses (to reduce focal pressure) and counseling patients on activity modification and weight avoidance on ulcerated feet. Unfortunately, studies show that even with good education, adherence to prescribed offloading is often poor when removable devices are used; classic accelerometry research by Armstrong et al. (2003) revealed that patients wore their removable walkers for only ~28% of daily steps on average, removing them during most walking despite an active ulcer [59]. This non-adherence underscores why consensus now strongly favors non-removable offloading methods during ulcer healing to “force compliance” [60]. Overall, the trajectory of evidence has moved the field from describing offloading concepts to implementing validated solutions. Offloading is one of the few interventions proven to significantly improve DFU outcomes, and its optimization continues to be a research focus. Current international guidelines set explicit offloading targets (e.g., at least 30% reduction in peak pressure at the ulcer site) and recommend clinical use of devices like TCCs or irremovable walkers as first-line for plantar ulcers [51]. The convergence of biomechanics and technology in this arena holds great promise; widespread adoption of effective offloading, coupled with novel smart-monitoring approaches, could drastically reduce the incidence of avoidable foot ulcers in the years ahead.

### 4.4. Clinical Implications of Plantar Pressure

Elevated plantar pressure is a well-established risk factor for diabetic foot ulcers (DFUs), as prolonged or repetitive loading can damage soft tissues, leading to ulcer formation. Consequently, clinical guidelines emphasize offloading techniques such as pressure-guided therapeutic footwear and custom orthotics, which have been shown to reduce ulcer recurrence rates by 46% to 65% [3]. Randomized trials confirm that improving therapeutic footwear based on plantar pressure measurements can reduce recurrence by about 46%. Recommended treatment thresholds suggest maintaining peak in-shoe plantar pressure below 200 kPa to decrease the risk of DFU recurrence [5]. Despite these findings, plantar pressure measurement still relies heavily on laboratory-based techniques, and remote monitoring solutions and standardized thresholds are still evolving. Additionally, only about 30% of ulcer locations correspond to peak pressure regions, indicating that shear stress, microcirculation, and neuropathy also play critical roles in ulcer formation. In our co-occurrence analysis, “plantar pressure” appears frequently but exhibits low centrality, indicating its widespread recognition in biomechanical studies but limited integration into broader clinical management frameworks. The low centrality highlights a clinical challenge: while plantar pressure is a key factor in DFU pathophysiology, it has not been fully incorporated into multidimensional management strategies. Therefore, future research should focus on combining plantar pressure measurements with comprehensive risk assessments, including neuropathy screening, vascular evaluation, and glycemic control, to refine risk stratification. Furthermore, integrating new evidence on pressure thresholds and offloading interventions into clinical practice guidelines is crucial [37]. Expanding interdisciplinary collaborations, particularly with experts in vascular medicine, wound healing, and regenerative therapies, will help link plantar pressure data with microcirculation, tissue perfusion, and healing outcomes, leading to more personalized and effective DFU care strategies. Despite these encouraging results, regenerative therapies are still in the early stages; sample sizes are small, and heterogeneity in cell sources, delivery methods and patient selection limits generalization. Growth factor therapy (e.g., becaplermin) improves cell recruitment and angiogenesis but suffers from low systemic bioavailability and safety concerns such as tumor promotion. Thus, clinicians should view these interventions as adjunctive options for patients with non-healing DFUs who have failed standard care, preferably within clinical trial settings. Developers may focus on improving the scalability and safety of cell-based products (e.g., allogeneic stem cells, exosome-based therapies) and designing biomaterials (scaffolds, hydrogels) that deliver cells or growth factors efficiently and sustainably. Collaboration with regulatory bodies and adherence to good manufacturing practice are essential to translate these therapies into routine practice.

### 4.5. Gaps in Clinical Translation and Interdisciplinary Integration

Despite substantial progress in understanding DFU pathophysiology and in developing effective interventions, significant gaps persist in translating this knowledge into improved population outcomes. One major challenge is achieving widespread implementation of evidence-based practices. Research has repeatedly shown that many patients do not receive optimal DFU care in real-world settings, even in high-income countries. For example, international surveys reveal a concerning discrepancy between offloading guidelines and actual practice—many clinics still rely on suboptimal methods (or patients’ normal shoes) for ulcer offloading, and adherence to using devices is low [52]. As mentioned, even when removable walkers are provided, patients often fail to wear them consistently, undermining their efficacy [59]. There is a clear need for improved patient engagement and education to address behavioral barriers to DFU prevention. Psychosocial factors such as depression, health literacy, and risk perception can significantly affect foot self-care and adherence [60]. Unfortunately, studies indicate that a large fraction of patients with healed ulcers do not appreciate their high risk of recurrence and thus may not rigorously adhere to preventive measures [58]. Targeted interventions like motivational interviewing and peer support are being explored to improve patient compliance, though results so far have been mixed for secondary prevention [55]. In addition, provider behavior and system-level factors contribute to gaps. A systematic review by Musuuza et al. (2020) found that while multidisciplinary team (MDT) care is associated with major amputation reductions in ~94% of studies, not all health systems have established dedicated foot care teams [61]. Many regions lack podiatrists or vascular specialists, and referrals to foot clinics are sometimes delayed until ulcers are advanced. Another translational gap lies in the uptake of new technologies. While smart insoles, remote temperature monitors, and telemedicine-based foot surveillance have proven benefits, their use in routine practice is not yet widespread [37,62]. Ensuring that payers recognize these long-term benefits and support coverage for preventive interventions is an ongoing policy challenge. There are also gaps in translating research findings into clinical guidelines and then into practice. Although international guidelines exist (IWGDF) and are updated regularly, their recommendations often take years to trickle into general practice. Some clinicians may not be aware of the latest evidence or may lack training in specialized techniques like total contact casting. This points to a need for broader dissemination of education—not only to specialists but to frontline providers (e.g., family physicians, diabetes educators) who can champion foot screenings and early referrals. On a related note, patient awareness is lacking; many patients with diabetes do not recall being counseled about foot care or properly screened for neuropathy [36]. The concept of “extended care” needs to be better translated into standard practice to break the cycle of recurrence. In conclusion, while the scientific community has made tremendous strides in identifying how to prevent and heal DFUs, the challenge now is implementation. Multidisciplinary team care, evidence-based offloading, advanced therapies, and patient engagement strategies all exist, but ensuring every high-risk patient can benefit from them is an ongoing struggle. Closing these gaps will likely require health system changes—investing in limb salvage teams, improving care coordination, and covering preventive interventions—as well as continued research into practical delivery models. The formation of diabetic foot centers of excellence and national limb preservation programs (as seen in some countries) are positive steps in this direction [63]. Additionally, fostering greater interdisciplinary integration—bringing together endocrinologists, surgeons, podiatrists, vascular specialists, wound nurses, and rehabilitation experts—is key to managing the multifaceted nature of DFUs [19]. The literature clearly indicates that such integrated approaches yield superior outcomes [38,61]. To better contextualize our findings, we compared this study with previous bibliometric analyses in the broader field of wound care and DFU. Some studies focused on the overall landscape of DFU research but primarily highlighted the dominance of infection and pharmacological treatments [64,65]. In contrast, our study reveals that plantar pressure management has evolved as a distinct, high-growth trajectory, particularly in the last five years. Furthermore, while some analyses showcased the maturity of surgical technologies, our findings highlight a nascent but rapidly expanding focus on regenerative medicine and smart wearable sensors in the context of pressure redistribution [11,66]. This specificity allows us to pinpoint a critical ‘translational gap’—the low centrality of plantar pressure despite its high publication frequency—which has not been explicitly identified in broader wound care bibliometrics.

### 4.6. Limitation

Because plantar pressure in DFU care is inherently embedded within multidisciplinary management pathways, the retrieved corpus may include studies primarily framed around wound care, infection control, vascular assessment/intervention, or rehabilitation, yet still containing substantively relevant plantar loading/offloading content. This multidisciplinary overlap can blur topic boundaries and may introduce heterogeneity in how plantar pressure is defined, measured, and discussed across disciplines.

The inclusion of the broad search term “pressure” was intended to increase retrieval sensitivity and capture studies addressing pressure redistribution and offloading strategies relevant to diabetic foot management. However, this approach may also have retrieved some records whose primary focus lies outside direct plantar pressure measurement.

In addition, reliance on a single bibliographic database limits cross-disciplinary coverage; multi-database bibliometric integration (e.g., WoSCC combined with Scopus, PubMed/MEDLINE, and engineering-focused databases such as IEEE Xplore) is needed to provide a more comprehensive map of plantar pressure research spanning biomechanics, engineering, podiatry, and clinical care. Despite manual curation and thesaurus-based harmonization, residual preprocessing errors (e.g., malformed low-frequency keywords) may persist and could affect the interpretation of marginal terms. Reproducibility of bibliometric preprocessing may also be influenced by threshold choices and manual normalization decisions, particularly for sparse network regions and low-frequency keywords, which may alter network topology, cluster boundaries, and the apparent prominence of emerging themes. Bibliometric indicators such as publication counts, citation frequency, and H-index primarily reflect research visibility and citation impact within the scientific literature rather than intrinsic research quality or clinical superiority.

This bibliometric analysis focused solely on the Web of Science Core Collection (WoSCC), which may limit the inclusion of relevant studies from other databases such as PubMed and Scopus. The WoSCC was chosen for its rigorous indexing of high-quality, peer-reviewed journals, ensuring reliable results. This exclusion may miss studies from non-English sources or those not indexed in the WoSCC. Future research could expand the database scope to provide a more comprehensive view of the literature. We presented trend predictions based on polynomial regression models, which aim to reflect the historical growth pattern in publication volumes.

Future research could integrate multiple databases using bibliometric merge tools (KKU-BiblioMerge) to provide a more comprehensive and objective analysis. Meanwhile, we acknowledge that this model assumes that past trends will continue in the future, which may not always be the case due to various unpredictable factors, such as shifts in research funding, emerging scientific interests, or changes in publication practices.

## 5. Conclusions

Our study presents a comprehensive bibliometric analysis of research between 2000 and 2024 on plantar pressure and diabetic foot ulcers (DFUs). DFUs are a severe complication of diabetes, often leading to infection, amputation, and reduced quality of life. Abnormal plantar pressure is a critical factor in ulcer formation and recurrence. Factors like neuropathy, muscle atrophy, and foot deformities cause localized pressure in areas such as the forefoot and hallux, leading to elevated peak pressures and shear stress, which exacerbate tissue damage. The manuscript reviews studies showing that higher pressures, especially in the forefoot, increase the risk of ulcers. Additionally, shear stress at common ulcer sites is implicated in DFU pathogenesis. Offloading interventions such as custom footwear, insoles, and total contact casting are highlighted as essential treatments to alleviate localized pressure and promote healing. However, despite these efforts, recurrence rates remain high, with experts advocating for continuous prevention strategies. Bibliometric trends indicate a growing focus on plantar pressure, with emerging themes like microcirculation, regenerative medicine, and smart wearable sensors. However, plantar pressure, despite its frequency in publications, remains underrepresented in broader research networks. The authors suggest future research should focus on integrating biomechanics with personalized care, expanding offloading innovations, and implementing long-term monitoring to reduce recurrence and amputation risks.

## Figures and Tables

**Table 1:** Top 20 Web of Science subject categories by number of publications.

Rank	Web of Science Categories	Record Count	Rank	Web of Science Categories	Record Count
**1**	Surgery	522	11	Cell Biology	61
**2**	Dermatology	501	12	Sport Sciences	61
**3**	Endocrinology Metabolism	304	13	Engineering Electrical Electronic	44
**4**	Orthopedics	241	14	Instruments Instrumentation	42
**5**	Medicine General Internal	176	15	Health Care Sciences Services	40
**6**	Engineering Biomedical	146	16	Multidisciplinary Sciences	37
**7**	Medicine Research Experimental	136	17	Biophysics	36
**8**	Nursing	87	18	Rehabilitation	34
**9**	Peripheral Vascular Disease	77	19	Chemistry Analytical	33
**10**	Pharmacology Pharmacy	67	20	Neurosciences	32

**Table 2:** Top 10 countries/regions by publication volume.

Rank	Country	NP	NC	AC	H-Index
**1**	USA	678	41,106	60.63	89
**2**	United Kingdom	308	20,229	65.68	66
**3**	China	285	5462	19.16	39
**4**	Netherlands	118	8379	54.01	40
**5**	Italy	114	3495	30.66	30
**6**	Australia	111	4224	38.05	35
**7**	India	102	1332	13.06	19
**8**	Germany	102	4012	39.33	35
**9**	Canada	77	4406	57.22	30
**10**	Spain	69	1272	18.43	19

**Table 3:** Top 10 authors by publication volume.

Rank	Author	NP	NC	AC	H-Index
**1**	Armstrong, David	76	12,576	165.47	38
**2**	Bus, Sicco A.	52	5304	102.00	29
**3**	Lavery, Lawrence A.	40	3407	85.18	25
**4**	Boulton, Andrew J. M.	39	5782	148.26	23
**5**	Lazaro-Martinez, Jose Luis	28	282	10.07	11
**6**	Chockalingam, Nachiappan	24	375	15.63	11
**7**	Lazzarini, Peter Anthony	22	797	36.23	15
**8**	Jan, Yih-Kuen	21	378	18.00	12
**9**	Van Netten, Jaap J.	20	788	39.40	13
**10**	Lipsky, Benjamin A.	18	5137	285.39	15

**Table 4:** Top 10 institutions by publication volume.

Rank	Organization	NP	NC	AC	H-Index
**1**	UNIV AMSTERDAM	68	6774	99.62	32
**2**	UNIV MIAMI	46	5731	124.59	25
**3**	UNIV WASHINGTON	43	6719	156.26	27
**4**	UNIV MANCHESTER	43	5753	133.79	21
**5**	UNIV ARIZONA	35	4755	135.86	19
**6**	QUEENSLAND UNIV TECHNOL	34	1561	45.91	20
**7**	ROSALIND FRANKLIN UNIV MED & SCI	33	4282	129.76	19
**8**	UNIV ILLINOIS	30	947	31.57	16
**9**	MANCHESTER ROYAL INFIRM	29	3998	137.86	19
**10**	UNIV COMPLUTENSE MADRID	27	285	10.56	11

**Table 5:** Top 10 source journals by publication volume and top 10 cited journals by citation frequency.

Rank	Journals	NP	Country	IF (JCR2023)	Cited Journals	NC	Country	IF (JCR2023)
**1**	JOURNAL OF WOUND CARE	115	United Kingdom	1.5	DIABETES CARE	7044	USA	14.8
**2**	INTERNATIONAL WOUND JOURNAL	79	United Kingdom	2.6	WOUND REPAIR REGEN	2434	USA	3.8
**3**	WOUNDS-A COMPENDIUM OF CLINICAL RESEARCH AND PRACTICE	73	USA	1.4	DIABETES-METAB RES	2374	United Kingdom	4.6
**4**	INTERNATIONAL JOURNAL OF LOWER EXTREMITY WOUNDS	53	USA	1.5	DIABETIC MED	2210	United Kingdom	3.2
**5**	WOUND REPAIR AND REGENERATION	47	USA	3.8	INT WOUND J	1991	United Kingdom	2.6
**6**	JOURNAL OF THE AMERICAN PODIATRIC MEDICAL ASSOCIATION	45	USA	0.5	J VASC SURG	1772	USA	3.9
**7**	DIABETES CARE	34	USA	14.8	J WOUND CARE	1641	United Kingdom	1.5
**8**	DIABETIC MEDICINE	34	United Kingdom	3.2	PLAST RECONSTR SURG	1287	USA	3.2
**9**	OSTOMY WOUND MANAGEMENT	34	USA	0	J AM PODIAT MED ASSN	1155	USA	0.5
**10**	ADVANCES IN SKIN & WOUND CARE	33	USA	1.7	DIABETOLOGIA	1127	Germany	8.4

**Table 6:** Burst keywords. The black bars represent the time period during which each keyword exhibited a citation burst, with darker/filled segments indicating the active burst duration and lighter segments indicating non-burst periods across the timeline (2000–2024).

Keywords	Year	Strength	Begin	End	2000–2024
**neuropathy**	2000	16.1	**2000**	2008	▃▃▃▃▃▃▃▃▃▂▂▂▂▂▂▂▂▂▂▂▂▂▂▂▂
**ulcer**	2000	14.02	**2000**	2006	▃▃▃▃▃▃▃▂▂▂▂▂▂▂▂▂▂▂▂▂▂▂▂▂▂
**risk factors**	2000	8.87	**2000**	2007	▃▃▃▃▃▃▃▃▂▂▂▂▂▂▂▂▂▂▂▂▂▂▂▂▂
**foot ulceration**	2000	8.4	**2000**	2008	▃▃▃▃▃▃▃▃▃▂▂▂▂▂▂▂▂▂▂▂▂▂▂▂▂
**pressure**	2000	6.99	**2002**	2007	▂▂▃▃▃▃▃▃▂▂▂▂▂▂▂▂▂▂▂▂▂▂▂▂▂
**lower extremity amputation**	2003	6.55	**2003**	2016	▂▂▂▃▃▃▃▃▃▃▃▃▃▃▃▃▃▂▂▂▂▂▂▂▂
**total contact cast**	2004	8.26	**2004**	2013	▂▂▂▂▃▃▃▃▃▃▃▃▃▃▂▂▂▂▂▂▂▂▂▂▂
**trial**	2001	9.1	**2007**	2015	▂▂▂▂▂▂▂▃▃▃▃▃▃▃▃▃▂▂▂▂▂▂▂▂▂
**multicenter**	2001	5.68	**2009**	2017	▂▂▂▂▂▂▂▂▂▃▃▃▃▃▃▃▃▃▂▂▂▂▂▂▂
**biomechanics**	2011	6.48	**2011**	2016	▂▂▂▂▂▂▂▂▂▂▂▃▃▃▃▃▃▂▂▂▂▂▂▂▂
**gait**	2010	5.36	**2013**	2019	▂▂▂▂▂▂▂▂▂▂▂▂▂▃▃▃▃▃▃▃▂▂▂▂▂
**randomized controlled trial**	2010	7.65	**2015**	2018	▂▂▂▂▂▂▂▂▂▂▂▂▂▂▂▃▃▃▃▂▂▂▂▂▂
**growth factors**	2017	5.75	**2017**	2019	▂▂▂▂▂▂▂▂▂▂▂▂▂▂▂▂▂▃▃▃▂▂▂▂▂
**iwgdf guidance**	2018	11.33	**2018**	2020	▂▂▂▂▂▂▂▂▂▂▂▂▂▂▂▂▂▂▃▃▃▂▂▂▂
**debridement**	2019	8.26	**2019**	2022	▂▂▂▂▂▂▂▂▂▂▂▂▂▂▂▂▂▂▂▃▃▃▃▂▂
**microcirculation**	2001	7.63	**2019**	2021	▂▂▂▂▂▂▂▂▂▂▂▂▂▂▂▂▂▂▂▃▃▃▂▂▂
**custom made footwear**	2019	6.07	**2019**	2020	▂▂▂▂▂▂▂▂▂▂▂▂▂▂▂▂▂▂▂▃▃▂▂▂▂
**exercise**	2020	6.49	**2020**	2021	▂▂▂▂▂▂▂▂▂▂▂▂▂▂▂▂▂▂▂▂▃▃▂▂▂
**footwear**	2020	5.97	**2020**	2021	▂▂▂▂▂▂▂▂▂▂▂▂▂▂▂▂▂▂▂▂▃▃▂▂▂
**wound care**	2021	9.09	**2021**	2024	▂▂▂▂▂▂▂▂▂▂▂▂▂▂▂▂▂▂▂▂▂▃▃▃▃
**insole**	2011	7.66	**2021**	2024	▂▂▂▂▂▂▂▂▂▂▂▂▂▂▂▂▂▂▂▂▂▃▃▃▃
**ischemia**	2013	6.91	**2021**	2024	▂▂▂▂▂▂▂▂▂▂▂▂▂▂▂▂▂▂▂▂▂▃▃▃▃
**epidemiology**	2022	8.17	**2022**	2024	▂▂▂▂▂▂▂▂▂▂▂▂▂▂▂▂▂▂▂▂▂▂▃▃▃
**wound dressing**	2022	6.95	**2022**	2024	▂▂▂▂▂▂▂▂▂▂▂▂▂▂▂▂▂▂▂▂▂▂▃▃▃
**outcm**	2020	5.93	**2022**	2024	▂▂▂▂▂▂▂▂▂▂▂▂▂▂▂▂▂▂▂▂▂▂▃▃▃

**Table 7:** Citation bursts of references. The black bars represent the time period during which each keyword exhibited a citation burst, with darker/filled segments indicating the active burst duration and lighter segments indicating non-burst periods across the timeline (2000–2024).

References	Year	Strength	Begin	End	2000–2024
**Armstrong DG, 2001, DIABETES CARE, V24, P1019, DOI 10.2337/diacare.24.6.1019, DOI **[15]	2001	14.22	**2002**	2006	▂▂▃▃▃▃▃▂▂▂▂▂▂▂▂▂▂▂▂▂▂▂▂▂▂
**Armstrong DG, 2005, DIABETES CARE, V28, P551, DOI 10.2337/diacare.28.3.551, DOI **[12]	2005	15.08	**2005**	2010	▂▂▂▂▂▃▃▃▃▃▃▂▂▂▂▂▂▂▂▂▂▂▂▂▂
**Katz IA, 2005, DIABETES CARE, V28, P555, DOI 10.2337/diacare.28.3.555, DOI **[16]	2005	11.95	**2005**	2010	▂▂▂▂▂▃▃▃▃▃▃▂▂▂▂▂▂▂▂▂▂▂▂▂▂
**Armstrong DG, 2005, LANCET, V366, P1704, DOI 10.1016/S0140-6736(05)67695-7, DOI **[17]	2005	20.78	**2006**	2010	▂▂▂▂▂▂▃▃▃▃▃▂▂▂▂▂▂▂▂▂▂▂▂▂▂
**Singh N, 2005, JAMA-J AM MED ASSOC, V293, P217, DOI 10.1001/jama.293.2.217, DOI **[3]	2005	16.92	**2006**	2010	▂▂▂▂▂▂▃▃▃▃▃▂▂▂▂▂▂▂▂▂▂▂▂▂▂
**Blume PA, 2008, DIABETES CARE, V31, P631, DOI 10.2337/dc07-2196, DOI **[13]	2008	26.45	**2008**	2013	▂▂▂▂▂▂▂▂▃▃▃▃▃▃▂▂▂▂▂▂▂▂▂▂▂
**Bus SA, 2008, DIABETES-METAB RES, V24, PS162, DOI 10.1002/dmrr.850, DOI **[18]	2008	15.12	**2009**	2013	▂▂▂▂▂▂▂▂▂▃▃▃▃▃▂▂▂▂▂▂▂▂▂▂▂
**Lipsky BA, 2012, CLIN INFECT DIS, V54, P1679, DOI 10.1093/cid/cis460, DOI **[19]	2012	19.99	**2013**	2017	▂▂▂▂▂▂▂▂▂▂▂▂▂▃▃▃▃▃▂▂▂▂▂▂▂
**Bakker K, 2012, DIABETES-METAB RES, V28, P225, DOI 10.1002/dmrr.2253, DOI **[20]	2012	11.85	**2013**	2017	▂▂▂▂▂▂▂▂▂▂▂▂▂▃▃▃▃▃▂▂▂▂▂▂▂
**Bus SA, 2013, DIABETES CARE, V36, P4109, DOI 10.2337/dc13-0996, DOI **[21]	2013	10.99	**2014**	2018	▂▂▂▂▂▂▂▂▂▂▂▂▂▂▃▃▃▃▃▂▂▂▂▂▂
**Bus SA, 2016, DIABETES-METAB RES, V32, P99, DOI 10.1002/dmrr.2702, DOI **[22]	2016	19.51	**2016**	2021	▂▂▂▂▂▂▂▂▂▂▂▂▂▂▂▂▃▃▃▃▃▃▂▂▂
**Bus SA, 2016, DIABETES-METAB RES, V32, P25, DOI 10.1002/dmrr.2697, DOI **[23]	2016	14.32	**2016**	2021	▂▂▂▂▂▂▂▂▂▂▂▂▂▂▂▂▃▃▃▃▃▃▂▂▂
**Rice JB, 2014, DIABETES CARE, V37, P651, DOI 10.2337/dc13-2176, DOI **[24]	2014	12.14	**2016**	2019	▂▂▂▂▂▂▂▂▂▂▂▂▂▂▂▂▃▃▃▃▂▂▂▂▂
**Frykberg RG, 2015, ADV WOUND CARE, V4, P560, DOI 10.1089/wound.2015.0635, DOI **[25]	2015	11.82	**2017**	2020	▂▂▂▂▂▂▂▂▂▂▂▂▂▂▂▂▂▃▃▃▃▂▂▂▂
**Schaper NC, 2016, DIABETES-METAB RES, V32, P7, DOI 10.1002/dmrr.2695, DOI **[14]	2016	11.82	**2017**	2020	▂▂▂▂▂▂▂▂▂▂▂▂▂▂▂▂▂▃▃▃▃▂▂▂▂
**van Netten JJ, 2016, DIABETES-METAB RES, V32, P84, DOI 10.1002/dmrr.2701, DOI **[26]	2016	11.61	**2017**	2021	▂▂▂▂▂▂▂▂▂▂▂▂▂▂▂▂▂▃▃▃▃▃▂▂▂
**Armstrong DG, 2017, NEW ENGL J MED, V376, P2367, DOI 10.1056/NEJMra1615439, DOI **[2]	2017	53.64	**2018**	2022	▂▂▂▂▂▂▂▂▂▂▂▂▂▂▂▂▂▂▃▃▃▃▃▂▂
**Liu S, 2017, THER CLIN RISK MANAG, V13, P0, DOI 10.2147/TCRM.S131193, DOI **[27]	2017	13.28	**2018**	2022	▂▂▂▂▂▂▂▂▂▂▂▂▂▂▂▂▂▂▃▃▃▃▃▂▂
**Jeffcoate WJ, 2016, LANCET DIABETES ENDO, V4, P781, DOI 10.1016/S2213-8587(16)30012-2, DOI **[28]	2016	11.38	**2018**	2021	▂▂▂▂▂▂▂▂▂▂▂▂▂▂▂▂▂▂▃▃▃▃▂▂▂
**Zhang PZ, 2017, ANN MED, V49, P106, DOI 10.1080/07853890.2016.1231932, DOI **[1]	2017	22.88	**2019**	2022	▂▂▂▂▂▂▂▂▂▂▂▂▂▂▂▂▂▂▂▃▃▃▃▂▂
**Schaper NC, 2020, DIABETES-METAB RES, V36, P0, DOI 10.1002/dmrr.3266, DOI **[29]	2020	27.28	**2021**	2024	▂▂▂▂▂▂▂▂▂▂▂▂▂▂▂▂▂▂▂▂▂▃▃▃▃
**Bus SA, 2020, DIABETES-METAB RES, V36, P0, DOI 10.1002/dmrr.3269, DOI **[30]	2020	19.22	**2021**	2024	▂▂▂▂▂▂▂▂▂▂▂▂▂▂▂▂▂▂▂▂▂▃▃▃▃
**Bus SA, 2020, DIABETES-METAB RES, V36, P0, DOI 10.1002/dmrr.3274, DOI **[31]	2020	11.98	**2021**	2024	▂▂▂▂▂▂▂▂▂▂▂▂▂▂▂▂▂▂▂▂▂▃▃▃▃
**Everett E, 2018, ANN NY ACAD SCI, V1411, P153, DOI 10.1111/nyas.13569, DOI **[32]	2018	11.8	**2021**	2024	▂▂▂▂▂▂▂▂▂▂▂▂▂▂▂▂▂▂▂▂▂▃▃▃▃
**Armstrong DG, 2020, J FOOT ANKLE RES, V13, P0, DOI 10.1186/s13047-020-00383-2, DOI **[33]	2020	13.69	**2022**	2024	▂▂▂▂▂▂▂▂▂▂▂▂▂▂▂▂▂▂▂▂▂▂▃▃▃

## Data Availability

The raw data supporting the conclusions of this article will be made available by the authors upon request since the data used in this study are publicly accessible from Web of Science using our search strategy.
